# Progress in the Preclinical and Clinical Study of Resveratrol for Vascular Metabolic Disease

**DOI:** 10.3390/molecules27217524

**Published:** 2022-11-03

**Authors:** Dongxiao Fan, Chenshu Liu, Zhongyu Zhang, Kan Huang, Tengyao Wang, Sifan Chen, Zilun Li

**Affiliations:** 1 Division of Vascular Surgery, The First Affiliated Hospital of Sun Yat-sen University, Guangzhou 510080, China; 2 National-Guangdong Joint Engineering Laboratory for Diagnosis and Treatment of Vascular Diseases, The First Affiliated Hospital of Sun Yat-sen University, Guangzhou 510080, China; 3 Guangdong Provincial Key Laboratory of Malignant Tumor Epigenetics and Gene Regulation, Guangdong-Hong Kong Joint Laboratory for RNA Medicine, Sun Yat-sen Memorial Hospital, Sun Yat-sen University, Guangzhou 510120, China; 4 Medical Research Center, Sun Yat-sen Memorial Hospital, Sun Yat-sen University, Guangzhou 510120, China

**Keywords:** resveratrol, vascular metabolic disease, vascular metabolism

## Abstract

Vascular metabolic dysfunction presents in various diseases, such as atherosclerosis, hypertension, and diabetes mellitus. Due to the high prevalence of these diseases, it is important to explore treatment strategies to protect vascular function. Resveratrol (RSV), a natural polyphenolic phytochemical, is regarded as an agent to regulate metabolic pathways. Many studies have proven that RSV has beneficial effects on improving metabolism in endothelial cells (ECs) and vascular smooth muscle cells (VSMCs), which provide new directions to treat vascular metabolic diseases. Herein, we overviewed that RSV could regulate cell metabolism activity by inhibiting glucose uptake, suppressing glycolysis, preventing cells from fatty acid-related damages, reducing lipogenesis, increasing fatty acid oxidation, enhancing lipolysis, elevating uptake and synthesis of glutamine, and increasing NO release. Furthermore, in clinical trials, although the results from different studies remain controversial, we proposed that RSV had better therapeutic effects at high concentrations and for patients with metabolic disorders.

## 1. Introduction

The rising prevalence of obesity and diabetes mellitus in the world leads to various metabolic disorders, among which vascular dysfunction is one of the major complications [1]. When the vascular homeostasis is perturbed, the endothelial cells’ function, vascular metabolism, and vasodilation would be affected, subsequently leading to vascular metabolic diseases that include atherosclerosis, hypertension, and peripheral arterial diseases, to name but a few [2]. Vascular metabolic diseases were regarded as vascular metabolic dysfunction-caused diseases or metabolic disease-induced vascular complications. In recent years, small molecule compounds have drawn much attention because of numerous advantages, such as non-invasion and stable drug concentration, for treating these diseases.

Polyphenolic phytochemicals have various pharmacological effects. For example, apigenin and naringin were identified as the drugs for treating diabetes, Alzheimer’s disease, depression, and cancer [3,4]. Resveratrol (RSV), another potential therapeutic compound, has drawn much attention in recent decades. In the 1990s, researchers found that the intake of saturated fats of French people and their serum cholesterol concentration were similar to British or American people, but their cardiovascular disease mortality was much lower than that of British or American people, which is called the “French Paradox” [5]. It was speculated that moderate red wine consumption might be a reason. RSV, a polyphenolic compound obtained from grapes, was regarded as the major compound in red wine exerting a cardiovascular protective role. Many other studies also reported RSV’s multiple beneficial functions, such as anti-oxidant, anti-inflammatory, anti-obesity, and anti-diabetes effects [6]. From then on, RSV was regarded as a beneficial agent for human health and widely tested in clinical trials.

In recent studies of animal models and humans, RSV represented a potential modulator for vascular metabolic activities and had a beneficial effect on vascular metabolic diseases. However, few reviews focused on the metabolism-improving effects of RSV in vascular cells. Thus, in this review, we aimed to discuss the beneficial effects of RSV on metabolic activities in vascular cells, the underlying mechanisms, and the clinical use of RSV, in order to get a comprehensive understanding of this agent in treating vascular metabolic diseases. Here, we used “resveratrol”, “vascular metabolism”, and “vascular metabolic diseases” as keywords and performed the search in PubMed, Web of Science, and Science Direct. We selected the articles which were relevant to our topic and further filtered them by reading the full text.

## 2. The Protective Effects of RSV on Vasculature in Vascular Metabolic Diseases

### 2.1. Regulating Glucose Metabolism in Vascular Metabolic Diseases

The vasculature is mainly composed of endothelial cells (ECs), vascular smooth muscle cells (VSMCs), and extracellular matrix. The metabolism of glucose flux in ECs includes glycolysis and aerobic oxidation. Studies exploring the energy source in human umbilical vein endothelial cells (HUVECs) showed that 85% of adenosine triphosphate in ECs was produced by glycolysis [7]. Through this pathway, ECs could save more oxygen and deliver it to other organs and tissue through trans-endothelial transfer [8], and protect cells from oxidative stress damage by reducing the production of reactive oxygen species (ROS) [9]. Similar to ECs, VSMCs are also glycolysis-addicted [10]. The alteration of enzymes or end products related to glycolysis, such as hexokinase, pyruvate kinase, pyruvate, and lactate, affected the proliferation and viability of VSMCs [11,12,13]. For example, Lambert and colleagues found that in vascular remodeling diseases, glycolysis is increased in VSMCs by measuring the protein expression and activity of hexokinase 2, which could promote proliferation and suppress apoptosis [11]. It is worth noting that hyperactivity of glycolysis in VSMCs and proliferation of cells within the vessel wall are vital features for vascular metabolic diseases such as atherosclerosis [14]. Therefore, regulating the glucose metabolism of vascular cells is vital for maintaining vascular physiological function.

RSV is a potent agent for regulating glycolysis. The glycolysis feature of ECs and VSMCs under aerobic conditions made them comparable to the tumor cells [7]. Glycolysis provides energy and generates metabolic intermediates for tumor cellular biological activities such as proliferation and migration. Thus, anti-glycolytic cancer therapy is an effective method to suppress tumor growth [15]. In different tumor cell lines, RSV could reduce glucose uptake and metabolism as well as further inhibit cellular proliferation and migration by regulating the activity or expression of glycolysis-related enzymes.

**Figure 1:**
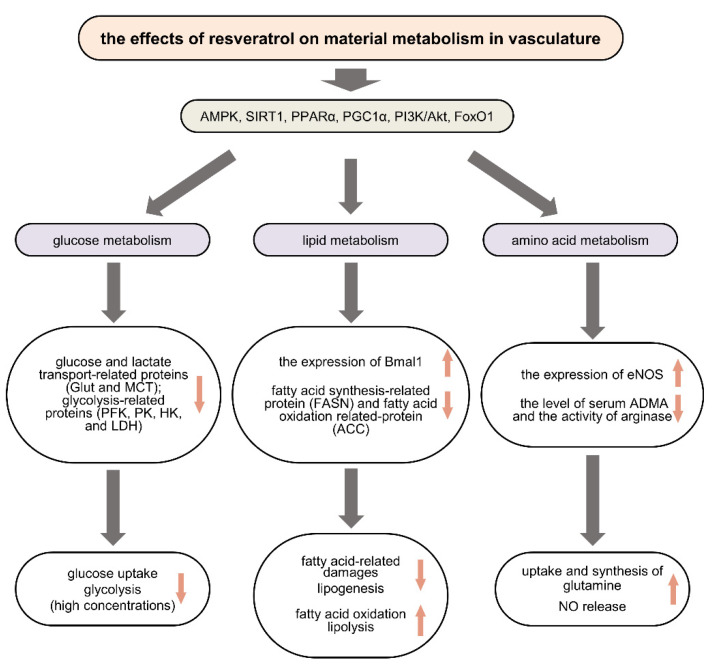
Resveratrol improves glucose, lipid, and amino acid metabolism in vascular cells by regulating different pathways and targets. Resveratrol reduces glucose uptake and glycolysis by inhibiting the expression of Glut, MCT, PFK, PK, HK, and LDH (**left**). Resveratrol improves fatty acid-related damages by upregulating the expression of Bmal1, decreases lipogenesis by suppressing the expression of FASN, and activates fatty acid oxidation by suppressing the expression of ACC (**middle**). Resveratrol increases the uptake and synthesis of glutamine. It also upregulates NO release by elevating the expression of eNOS, suppressing the level of serum ADMA, and inhibiting the activity of arginase (**right**). AMPK: AMP-activated protein kinase; SIRT1: silent information regulator 1; PPARα: peroxisome proliferator-activated receptor-gamma coactivator-1α; PGC1α: peroxisome proliferator-activated receptor-gamma coactivator-1α; PI3K: Phosphoinositide 3-kinase; FoxO1: forkhead box protein O1; Glut: glucose transporter; MCT: monocarboxylate transporter; PFK: 6-phosphofructo-1-kinase; PK: pyruvate kinase; HK: hexokinase; LDH: lactate dehydrogenase; Bmal1: brain and muscle arnt-like protein-1; FASN: fatty acid synthase; ACC: acetyl-CoA carboxylase; NOS: nitric oxide synthase; ADMA: Asymmetric dimethylarginine.

In some studies, the investigators focus on the effects of RSV on vascular glucose metabolism flux (Figure 1). In an experimental endometriosis model of Wistar rats, after administrating 40 mg/kg RVS orally, the glycolysis and neovascularization were inhibited by suppression of the expression levels of Glut-1, Glut-3, monocarboxylate transporter (MCT)-1, and MCT-4 in ectopic endometrial tissue [16]. Similarly, in HUVECs, RSV reduced the vascular endothelial growth factor-induced hyper levels of glycolysis-related mRNA and protein such as Glut1, 6-phosphofructokinase-1, pyruvate kinase M2, and hexokinase II in a dose-dependent manner [17].

RSV is a specific activator of silent information regulator 1 (SIRT1) (Figure 1). In a recent study, Huang et al. found that the RSV-mediated phosphorylated activation of SIRT1 is dependent on another enzyme, liver kinase B1 [18]. RSV promoted the binding of liver kinase B1 and SIRT1, and then liver kinase B1 phosphorylated SIRT1 at three residues (Ser615, Ser669, and Ser732). The phosphorylated SIRT1 enhanced the intramolecular interaction and further exerted deacetylase activity [18]. The effects of SIRT1 on glucose metabolism are on the transcriptional level. It promotes the deacetylation of many metabolic transcriptional regulators in vitro and in vivo [19,20]. Although the specific mechanism of SIRT1 maintaining glucose homeostasis in vascular cells remains elusive, it is deducible that SIRT1 decreased glycolysis via activating peroxisome proliferator-activated receptor-gamma coactivator-1α (PPARα) and suppressing hypoxia-inducible factor-1α (HIF-1α) in muscle, white adipose tissue, liver, and pancreas [21,22].

Interestingly, in diabetes mellitus model mice or hyperglycemic conditions, RSV could improve glucose metabolic disorders. In a recent study, Zheng and colleagues found that RSV (mixed with the powdered high-fat diet to a concentration of 0.2%, 18 days consecutively administration) reduced plasma glucose significantly in the high-fat diet-induced gestational diabetes mellitus mice model [23]. They also established an insulin-resistant adipocyte model and found that RSV (0.1 μM) enhanced glucose intake, miR-23a-3p expression, and PI3K/Akt pathway activation. Moreover, it was reported that RSV (10 μM) could improve hyperglycemia-induced endothelial dysfunction and enhance glycolysis by activating SIRT1-FoxO1-c-Myc pathway [24]. One of the possible reasons for the opposite regulating effects of RSV on glucose metabolism under different conditions is that RSV could enhance glucose uptake and metabolism at relatively low concentrations and had reversed the effects on glucose metabolism at relatively high concentrations. Although the regulation effect of RSV on glucose metabolism may be different at various concentrations, choosing the appropriate concentration can significantly improve glucose metabolism disorders under different pathological conditions. Thus, for treating glucose metabolism dysfunction in vascular metabolic diseases, RSV is a potent candidate.

### 2.2. Regulating Lipid Metabolism in Vascular Metabolic Diseases

Lipid metabolism in vasculatures is also vital for maintaining vascular homeostasis. The synthesis of fatty acids was reported to participate in the process of vessel sprouting, permeability, and endothelial nitric oxide synthase (eNOS) palmitoylation in ECs [25,26]. However, in vascular metabolic diseases, the transportation and metabolism of fatty acids are disrupted. It was reported that in pigment epithelial-derived factor (an important regulator in lipid metabolism) deficiency ApoE^−/−^ mice, the atherosclerotic plaque formation was aggravated and the capacity of fatty acid uptake was upregulated, which resulted in fatty acid accumulation in the peripheral tissues [27]. In in vitro experiments, the pigment epithelial-derived factor could downregulate the protein expression of fatty acid transport proteins 3/4, and protect HUVECs by reducing fatty acid uptake and accumulation. Palmitic acid (PA), a saturated long-chain fatty acid with a 16-carbon backbone and the first fatty acid produced during lipogenesis, is considered to have adverse effects on the metabolism of vasculature in adults. In ECs and VSMCs, PA stimulated the production of ROS and caused oxidative stress damage via the activation of protein kinase C-mediated nicotinamide adenine dinucleotide phosphate oxidation [28]. As well as PA, other fatty acids, such as stearic acid and linoleic acid, were reported to induce inflammation and insulin resistance by reducing the AMP-activated protein kinase (AMPK)/PI3K/Akt/eNOS pathway [29,30].

RSV could improve fatty acid-related damage (Figure 1). Two independent studies showed that RSV reverses the detrimental effects of PA by activating AMPK signal pathway in skeletal muscle [31,32]. Subsequently, Li et al. used a mixture of fatty acids (oleic acid: PA = 2:1) to induce glycolipid metabolic disorders in hepatocytes [33]. They found that RSV could ameliorate these disorders by upregulating the expression of brain and muscle arnt-like protein-1. In this study, when knocking down the brain and muscle arnt-like protein-1 by small interfering RNAs, RSV-stimulated phosphorylation and activation of AMPK was blunted. However, though a few studies reported similar protective effects of RSV in ECs or VSMCs, it remains necessary to further investigate the effects of RSV on fatty acid accumulation-induced vascular impairment.

In addition to fatty acid transportation and metabolism dysfunction, other lipid metabolism pathways are also perturbed in vascular metabolic diseases. It is reported that although a low dose of oxidized low-density lipoprotein (LDL) increased the efflux of cholesterol and the expression of the ATP-binding cassette subfamily A member 1 and ATP binding cassette subfamily G member 1 (two cholesterol efflux transport proteins) in endothelial cells, these alternations were reversed as the dose of oxidized LDL increased [34], which suggested that the function of cholesterol efflux was impaired in cells. To date, there is a lack of direct evidence that RSV improves lipid metabolism in vascular cells, but RSV could regulate lipid metabolism in lipid metabolic tissues such as adipose tissue and liver, which might reduce plasma lipids and the adverse effects of lipids on vessel walls in the body. In the studies of adipocytes, RSV decreased lipogenesis, promoted lipolysis, and inhibited preadipocyte differentiation, which contributed to reducing lipid accumulation [35,36,37]. In hepatocytes, RSV suppressed lipid accumulation through a SIRT1-mediated decrease in fatty acid synthesis by elevating phosphorylation and inhibiting the activity of acetyl-CoA carboxylase (a de novo fatty acid synthesis-related enzyme) and downregulating the protein expression of fatty acid synthase [38]. RSV is also a potent agent to regulate plasma lipid components. In the KKAy mice, a model of obesity and metabolic disorders, RSV could significantly reduce plasma triglyceride (TG), fatty acid, and malonaldehyde, and increase high-density lipoprotein cholesterol (HDL-C) and superoxide dismutase. More importantly, these effects are dose-dependent [39].

RSV could elevate fatty acid oxidation via SIRT1-mediated activation of PPARα and its coactivator peroxisome proliferator-activated receptor-gamma coactivator-1α (PGC1α) [40], which made cells switch to the hypermetabolic state. However, VSMCs are prone to proliferate at a hypermetabolic state, which is identified in several pathological situations such as vascular damage, vascular inflammation, and metabolic stresses [41]. Interestingly, many studies presented that RSV suppressed VSMC proliferation [42,43]. In in vivo experiments, RSV inhibited intimal hyperplasia in a wire-injured femoral artery mouse model by a heme oxygenase-1-dependent pathway [44]. The underlying mechanism of this contradiction needs to be further explored. In conclusion, although RSV has benefits on lipid metabolism in vascular metabolic diseases, such as atherosclerosis by reducing serum lipid profiles and improving the lipid metabolic pathways in the liver and adipose tissue, the direct effects and mechanism of RSV on lipid metabolism in ECs/VSMCs remain elusive. Thus, future studies should focus more on the effects of RSV on disrupted lipid metabolism in vascular cells.

### 2.3. Regulating Amino Acid Metabolism in Vascular Metabolic Diseases

Amino acids and amino acid metabolism are also vital for the vascular cell biological process. Glutamine is the most abundant non-essential amino acid in circulation and the effects of glutamine metabolism on vascular cell proliferation have been reported in many studies [45]. In ECs, glutamine deprivation or glutaminase 1 (an enzyme metabolizing glutamine to glutamate) deficiency could reduce protein synthesis, imbalance redox homeostasis, and inhibit the mechanistic target of the rapamycin (mTOR) signaling pathway, which might impair vessel sprouting [45]. In VSMCs, overexpressing the solute carrier family 1 member 5, a sodium-dependent amino acids transporter for multiple amino acids, could promote glutamine uptake, mTORC1 activation, and further enhance VSMC proliferation [46]. Arginine is the main source to produce NO which serves as a vascular protective molecule regulating vasodilation via inhibiting mitochondrial respiration [47]. It was reported that a low level of arginine in ECs might lead to ECs dysfunction and inhibition of arginase or supplementation of arginine could reverse ECs function [48,49,50]. The NOS, catalyzing arginine to NO, has different isoforms in different cell types. The main NOS is eNOS in ECs, while in VSMCs the NOS consists of eNOS, neuronal (nNOS), and inducible (iNOS) [51]. Therefore, regulating the activity of NOS is of great significance for arginine metabolism.

In a diabetic rat retina model, RSV could protect retinal function in high glucose-induced retinal dysfunction via elevating glutamate uptake, enhancing glutamine synthetase activity, and increasing glutamine synthetase and glutamate transporters expression [52]. RSV also regulated glutathione (a tripeptide composed of three amino acids including glutamate, cysteine, and glycine) metabolism in vascular cells. It is reported that RSV could increase the level of glutathione in the culture medium supernatant of VSMCs treated with high glucose, which enhanced cell total antioxidant capacity [53].

RSV affects the production of NO (Figure 1). It was reported that RSV could decrease the cigarette smoke-induced eNOS acetylation in HUVECs by activating SIRT1 and further lead to NO release which exerts vasoprotective and cardioprotective effects [54]. In another in vivo experiment, RSV could significantly elevate the expression of eNOS in the aorta of ApoE^−/−^ mice treated with high cholesterol diet, which contributed to the prevention of dyslipidemia-induced endothelial dysfunction and atherosclerosis [55]. Asymmetric dimethylarginine (ADMA) is an endogenous NOS inhibitor. It mainly exists in plasma to inhibit the production of NO in vessels [56]. Many studies reported the increased level of ADMA in different vascular metabolic diseases [57]. Interestingly, the result from a clinical double-blind randomized trial showed that RSV (1000 mg/day, 8 weeks) could significantly decrease the level of ADMA in patients with diabetes [58]. Moreover, RSV exhibited arginase inhibitory activity in VSMCs, which led to the accumulation of arginine and the augment of NO production in vascular cells [59]. Together, RSV could improve amino acid metabolism dysfunction and protect vasculatures from metabolic vascular diseases.

## 3. Clinical Trials of RSV in the Treatment of Vascular Metabolic Diseases

RSV is a potential agent for maintaining cell metabolic homeostasis. Thus, for the purpose of exploring the clinical transformation value of RSV, researchers performed a large number of clinical studies to confirm the therapeutic effects of RSV on vascular metabolic diseases. Here, these clinical researches will be reviewed as follows.

### 3.1. The Effect of RSV on Atherosclerosis

In preclinical research, RSV could modify the lipid profile by decreasing the serum TG and LDL, as well as increasing HDL in Wistar rats [60], streptozotocin-induced gestational diabetes model rats [61], KKAy mice [39], ApoE^−/−^ mice [62], high-fat-diet-fed mice [63], and healthy crossbred pigs [64] (Table S1). Thus, researchers performed various clinical trials to explore the lipid-lowering effects of RSV in different population cohorts (Table 1). However, the lipid-regulating effects of RSV in human subjects remain controversial. In a randomized double-blind crossover study of healthy obese human subjects, after being treated with RSV at 150 mg/day for 30 days, RSV significantly reduced the plasma TG level (2.16 ± 0.21 vs. 2.29 ± 0.23 mmol/L in RSV vs. placebo) and lowered intrahepatic lipid content compared to placebo [65]. Similarly, in another randomized, double-blind, crossover trial, RSV (500 mg/day) treatment for 30 days significantly reduced the level of plasma TG in healthy adult smokers [66]. In people at high risk for cardiovascular diseases and under statin treatment for prevention, RSV (8 mg/day, 6 months, in RSV-enriched grape extract) significantly decreased oxidized LDL by 20% and decreased LDL-C by 4.5% [67]. In a prospective randomized study, 57 patients with type 2 diabetes were divided into two groups (28 in the RSV intervention group, and 29 in the control group). After 3 months of RSV (250 mg/day) or a placebo supplement, plasma cholesterol (4.70 ± 0.90 vs. 4.33 ± 0.76 mmol/L) and LDL-C (2.58 ± 0.83 vs. 2.26 ± 0.65 mmol/L) reduced significantly in the RSV intervention group, while these parameters increased in the control group (4.89 ± 0.89 vs. 5.07 ± 0.90 mmol/L and 2.80 ± 0.80 vs. 2.98 ± 0.79 mmol/L) [68].

However, there are several studies indicating that RSV only altered a part of the lipid profiles. In the healthy smokers’ study mentioned above, it is noted that the TC level in plasma did not change. In the diabetes patients’ study mentioned above, the plasma TG and HDL-C did not change. Another study enrolling 50 patients with nonalcoholic fatty liver disease (NAFLD) showed that, although RSV supplementation (600 mg/day, 12 weeks) could significantly reduce body weight and body mass index, there were no significant changes in oxidized-LDL, LDL-C/HDL-C and LDL-C/oxidized-LDL [69]. Moreover, in a randomized double-blind study of 29 nonobese people with normal glucose tolerance (15 in the RSV intervention group, 14 in the placebo group), RSV supplementation (75 mg/day, 12 weeks) did not alter glucose, insulin, plasma lipids, and markers of inflammation [70]. Then, the researchers performed hyperinsulinemic-euglycemic clamp experiments in different metabolic organs. The result showed that insulin sensitivity in the liver, skeletal muscle, or adipose tissue did not change [70]. In accordance with this study, another study enrolled 48 healthy and slightly overweight subjects who were treated with 30 days of RSV (250 mg, twice a day) and caloric restriction (1000 cal/day), respectively. Compared to caloric restriction, RSV intervention did not reduce lipid profile (HDL-C, LDL-C, TG) but even increased total cholesterol subtly [71]. The authors concluded that 30 days of RSV supplement had no effect on lipid metabolism. One of the possible reasons is that RSV has little effect on the normal levels of glucose and lipid metabolism in relatively healthy individuals. In a meta-analysis of seven randomized controlled trials, the author concluded that RSV supplementation did not have effects on the lipid profile including TC, LDL-C, HDL-C, and TG [77]. Another meta-analysis of 21 randomized clinical trials published in 2018 showed that RSV could only significantly decrease TG (weighted mean difference: 0.58 mmol/L) but had no effect on TC, LDL-C, and HDL-C [78]. In 2022, a meta-analysis, consisting of 25 randomized controlled trials, demonstrated that RSV could regulate plasma lipid profiles [79]. There were a total of 1171 participants in the selected articles, which included 578 in the placebo group and 593 in the RSV intervention group. There was a significant decrease in TC (standard mean difference: −0.15) and LDL-C (standard mean difference: −0.42), and a significant increase in HDL-C (standard mean difference: 0.16) following RSV administration. However, there was no significant effect of RSV on TG. The results of these meta-analyses also indicated that RSV might have better plasma lipid-regulating effects in hyperlipidemic patients than that in patients with normal plasma lipids (might be lowered by statins or other drugs). Thus, it is important for the researchers to exclude the interference of other lipid-lowering drugs in future studies.

Some studies focused on patients diagnosed with atherosclerosis. In a randomized, prospective, and double-blind study, 214 patients with carotid stenosis >70% and in a surgical intervention request were randomly divided into two groups, including one group treated with one tablet of Cardioaspirin^®^ and one tablet of Aterofisiol^®^ (a combination of omega-3, vitamin K2, vitamin B6, vitamin B12, procyanidolic oligomers, and 20 mg RSV), and another group treated with Cardioaspirin^®^ and placebo. After being intervened for 25 days, patients underwent an endarterectomy to remove plaques for further investigation. The dry weight of lipid and cholesterol in removed plaques was significantly reduced (0.232 ± 0.018 vs. 0.356 ± 0.022; 0.036 ± 0.006 vs. 0.053 ± 0.007 mg/mg dry weight in Cardioaspirin^®^ and Aterofisiol^®^ group vs. Cardioaspirin^®^ and placebo group) after Aterofisiol^®^ treatment [72]. Furthermore, many studies reported the effects of RSV on coronary artery diseases (CAD) [73,74,75,76]. In a cohort of patients with type 2 diabetes mellitus and CAD, participants were treated with RSV (500 mg/day) or a placebo for 4 weeks. Compared to the placebo group, the plasma HDL-C was significantly increased (difference in the mean outcome measures between RSV and placebo groups: 3.38 mg/dL) in the RSV administration group, while the other lipids did not significantly alter between the two groups [73]. In a double-blind, randomized study, RSV (10 mg/day, 3 months) supplement reduced LDL-C significantly in patients with stable CAD. More importantly, RSV could significantly improve endothelial function by measuring flow-mediated dilation (FMD, a marker of endothelial function) [74]. However, in another triple-blind randomized study, the researchers reported that one year of RSV-containing grape extract supplement (grape phenolics plus 8 mg RSV for 6 months and 16 mg for the following 6 months) had no effect on most of the plasma lipid profiles. However, the level of an anti-inflammatory factor, plasma adiponectin, significantly increased. Thus, they proposed the therapeutic effects of RSV on CAD via inhibiting inflammation rather than lipid lowering [75]. Interestingly, some studies reported the acute supplementation of RSV could alleviate CAD [76]. A study reported that acute supplementation of a high dose of RSV (330 mg/day, 3 days) could significantly improve FMD in older CAD patients undergoing coronary artery bypass grafting but not in patients undergoing percutaneous coronary intervention [76]. The authors ascribed the contradiction to the difference in endothelium intactness after these two surgical revascularization methods.

Altogether, the effects of RSV on atherosclerosis patients included improving lipid metabolism, inhibiting inflammation, and enhancing endothelial function. However, due to the distinction of RSV dosage and metabolic disorder levels among different patient populations, the efficacy of RSV in different clinical trials varied. Thus, it is important to further optimize the dosage and the duration of RSV in large or multicenter clinical trials to investigate the usage of RSV in different populations.

### 3.2. The Effect of RSV on Hypertension

Hypertension is considered as another life-threatening vascular metabolic disease that is characterized by vascular dysfunction and injury. The antihypertensive effect of RSV has been proven in different animal hypertension models, including Angiotensin II-induced hypertension [80], partial nephrectomy hypertension model [81], DOCA-salt hypertension model [82], and early weaning induced hypertension [83], which led to many transformational studies in hypertension.

According to the preclinical results, many clinical studies were designed to explore the benefits of acute or chronic supplementary RSV on hypertension subjects (Table 2). In a cohort of cardiovascular disease patients and healthy individuals, arteries were isolated from their subcutaneous fat biopsies, and wire myography experiments (an experiment to test artery dilatory responses) were then performed. After treatment with RSV, rapid vasorelaxation was observed in a NO-dependent manner [84]. A similar conclusion was also obtained by a study group that used vascular rings derived from patients undergoing coronary artery bypass operation [85]. In other studies, the authors showed that one hour of RSV (30, 90, and 270 mg) treatment increased FMD response in a dose-dependent manner for healthy obese individuals [86] and hypertension patients [87] without changing artery diameter. In addition, long-term RSV administration also has benefits for controlling blood pressure. The same group conducted a study to investigate the effects of chronic RSV supplementation on FMD in healthy obese adults and found that RSV (75 mg/day, 6 weeks) induced a 23% increase in FMD [88]. In hypertension subjects, using a combination of isolated phytochemicals (containing 60 mg of RSV, 330 mg grape seed and skin extract, 100 mg green tea extract, and 60 mg a blend of quercetin, ginkgo biloba and bilberry) for 28 days reduced diastolic pressure significantly (reduced by 4.4 mm Hg), while diastolic blood pressure was not significantly affected [89]. There are two meta-analyses about the effects of RSV on blood pressure [90,91]. Both meta-analyses showed that the administration of RSV affects neither systolic nor diastolic blood pressure. However, subgroup analyses of one meta-analysis showed that RSV reduced systolic blood pressure at a high daily dosage (≥300 mg/day) [90]. It is speculated that low bioavailability might be one of the reasons explaining the positive effects of RSV at high concentrations. In the other meta-analysis, RSV demonstrated favorable, though not significant, blood pressure-lowering effects on systolic blood pressure, mean arterial pressure, and pulse pressure [91]. Moreover, the blood pressure-lowering effect of RSV is stronger in those with diabetes. Interestingly, both of the meta-analyses demonstrated that RSV had potential effects on systolic blood pressure rather than diastolic blood pressure. The underlying mechanism remains elusive. Thus, more clinical trials need to be conducted to clarify the effects of RSV on blood pressure. Novel methods used to enhance the efficacy of RSV in treating hypertension, such as combining RSV with other compounds [92] and using nanocarriers [93], are also worthy to be further developed.

### 3.3. The Effect of RSV on Ischemia

The protective effects of RSV against ischemic diseases have been reported in many preclinical studies including stroke [94], myocardial ischemia [95], and peripheral artery disease [96]. The possible mechanism was that RSV could protect ECs and reduce the damage and inflammation caused by ischemia in the blood vessel wall [97]. Accordingly, clinical studies were conducted to investigate the effects of RSV on patients with the ischemic disease (Table 3).

Until now, there have not been any clinical studies of RSV treatment alone for stroke patients. Nevertheless, RSV was reported to serve as an adjuvant with the recombinant tissue plasminogen activator (r-tPA) to improve patients’ outcomes. In this study, patients suffering from an ischemic stroke were divided into two cohorts depending on the treatment time interval after stroke onset (early onset-to-treatment time (OTT) group: treatment within 120 min after stroke onset; and delay OTT group: treatment between 120–240 min after stroke onset). Then, each cohort was randomized to two groups treated with r-tPA and r-tPA plus RSV, respectively. The results showed that co-administration of RSV and r-tPA could prolong the clinical therapeutic window of r-tPA, which is a promising therapeutic direction for patients receiving late stroke treatment [98]. Many clinical studies in healthy adults and stroke-free diabetes patients also showed that RSV increased the blood flow perfusion in cerebral vessels. In a double-blind crossover investigation, healthy adult subjects received a placebo and RSV (250 mg and 500 mg) and then performed cognitive tasks under the monitoring of near-infrared spectroscopy (a noninvasive brain imaging technique). Interestingly, treatment with RSV increased cerebral blood flow during task performance and the effect was dose-dependent [99].

The protective effects of RSV on myocardial infarction had mentioned above (in the atherosclerosis section). These studies concluded that RSV supplementation improved endothelial function and decreased the risk of secondary myocardial infarction [74,75]. Moreover, RSV also had protective effects on patients with stable angina pectoris [100]. This double-blind randomized study consisted of three groups treated with RSV (20 mg/day, 60 days), calcium fructoborate (CF, a natural compound, 112 mg/day, 60 days), and RSV plus CF, respectively. The results indicated that the level of high-sensitivity C reactive protein and N-terminal prohormone of brain natriuretic peptide significantly decreased (24.6% and 59.7% respectively) after RSV intervention, and the effects were enhanced by using the combination of RSV and CF.

The main causes of peripheral artery diseases are atherosclerosis and diabetes. Several clinical studies focus on the protective efficiency of RSV on peripheral artery diseases. In the RESTORE study, researchers determined a six-min walk distance as the primary outcome to evaluate blood perfusion of lower extremities in older patients with peripheral artery disease [101]. After 6 months of RSV (125 mg and 500 mg) supplementation, they drew a passive conclusion that RSV could not improve the walking performance in these patients, even after they decrease the statistical power (*p* < 0.1). Subsequently, some novel techniques provided promising prospects for the use of RSV to improve peripheral vessel lesions. The paclitaxel-RSV-matrix-coated peripheral balloon had been proven to benefit patients in the CONSEQUENT trial [102,103]. This trial allocated patients with femoropopliteal lesions into the paclitaxel-RSV-drug-coated balloon (DCB) group and plain old balloon angioplasty (POBA) group randomly. The researchers collected endpoint outcomes from the patients at 6 months, 1 year, and 2 years, and found that compared with POBA, DCB significantly reduced in-lesion late lumen loss at 6 months and target lesion revascularization at three periods. They also observed a significant increase in walking distance in the DCB group which indicates the beneficial effects of RSV on peripheral artery diseases. In conclusion, RSV is a potent regent for treating ischemia diseases. More large-scale clinical trials are needed to further verify the effects of RSV on patients with different ischemic diseases.

### 3.4. The Effect of RSV on Vascular Complications of Metabolic Disease

Metabolic diseases, including obesity, NAFLD, and diabetes, are independent risk factors for vascular metabolic disease. In previous preclinical studies, it was reported that RSV could modify the metabolic function and further protect blood vessels via regulating multiple pathways, including AMPK phosphorylation, SIRT1 activation, and decreasing ROS [104,105,106,107]. However, similar to the clinical studies mentioned above, although researchers had obtained promising results in in vivo and in vitro experiments, we cannot get a consistent conclusion about RSV efficiency from the existing clinical research (Table 4). Some clinical studies reported that RSV supplementation could improve glucolipid metabolism and reduce inflammation [108,109,110,111], while some studies found that these metabolic parameters did not change after intervention [112,113,114], as mentioned above. One study even reported that RSV had adverse effects on the subjects at a high concentration for overweight older adults (mentioned below).

In older patients with glucose intolerance, RSV was administrated at 2–3 g daily for 6 weeks. The reactive hyperemia peripheral arterial tonometry was used to determine vascular function. The results showed that RSV supplementation could significantly increase the reactive hyperemia index which indicated its potential to improve vascular function [115]. However, they failed to observe the alteration of gene expression related to glucose metabolism [115]. Similarly, in a double-blind randomized crossover trial, the fatty acid oxidation capacity did not alter after the RSV supplement (1000 mg/day, 4 weeks) in patients with very long-chain acyl-CoA dehydrogenase deficiency or carnitine palmitoyl transferase II deficiency [116]. In another double-blind randomized crossover trial, RSV (150 mg/day, 34 days) could not reduce arterial inflammation as measured with 18F-fluorodeoxyglucose PET in subjects at risk of developing type 2 diabetes mellitus [117]. Unfortunately, although the beneficial effects of RSV on CAD patients with potential dyslipidemia (mentioned in the previous section) and pediatric obese subjects [118] had been reported in some studies, some clinical studies about the effects of RSV on NAFLD or obese subjects’ vascular function showed passive results. These studies indicated that RSV supplementation had no effect on the levels of plasm-soluble adhesion molecules, inflammatory markers, and biomarkers of endothelial function [119,120,121]. However, the concentrations used in these trials were relatively low. Surprisingly, in a study, it was reported that RSV even significantly increased cardiovascular disease risk factors (such as intercellular adhesion molecule-1, vascular cell adhesion molecule-1, and oxidized-LDL) at a high concentration (1000 mg/day, 90 days) in overweight older adults [122]. The possible reason might be that the tissues were in the repair process during which pro-inflammatory processes and free radical formation are present [123]. The specific mechanism needs to be further explored.

## 4. Conclusions

RSV might have great potential for the treatment of vascular metabolic diseases from numerous preclinical studies. Although the results from different clinical trials remain controversial, we proposed that RSV had better therapeutic effects at high concentrations and for patients with metabolic disorders. Thus, it is still very important and urgent to identify the molecular mechanism of RSV in vessels wall, and perform larger and higher levels of clinical studies to further confirm the effects of RSV in patients with vascular metabolic diseases.

## Figures and Tables

**Table 1:** Summary of clinical trials involving the use of RSV in atherosclerosis.

Cohort (No.)	Dose and Duration of RSV	Main Outcome after RSV Administration	First Author, Year, Reference
Healthy, obese men (*n* = 11) treated with placebo and RSV	150 mg/day, 30 days	plasma TG ↓ (2.16 ± 0.21 vs. 2.29 ± 0.23 mmol/L in RSV vs. placebo); intrahepatic lipid content ↓; non-esterified fatty acids →.	Timmers, 2011 [65]
Healthy smokers (*n* = 50) treated with placebo and RSV	500 mg/day, 30 days	plasma TG ↓; plasma TC and HDL-C →.	Bo, 2013 [66]
Patients at risk for cardiovascular disease randomized into placebo (*n* = 25), grape extract (*n* = 25) and RSV-enriched grape extract (*n* = 25) groups	containing ~8 mg RSV/day, 6 months	plasma ox-LDL and LDL-C ↓ (decreasing oxidized-LDL by 20% and decreasing LDL-C by 4.5%); plasma TG, TC and HDL-C →.	Tomé-Carneiro, 2012 [67]
Type 2 diabetes mellitus patients randomized into control (*n* = 29) and RSV intervention (*n* = 28) groups	250 mg/day, 3 months	plasma TC and LDL-C ↓ (4.70 ± 0.90 vs. 4.33 ± 0.76 mmol/L and 2.58 ± 0.83 vs. 2.26 ± 0.65 mmol/L after RSV intervention, respectively); plasma TG and HDL-C →.	Bhatt, 2012 [68]
Patients with nonalcoholic fatty liver disease randomized into placebo (*n* = 25) and RSV (*n* = 25) groups	600 mg/day, 12 weeks	plasma ox-LDL, LDL-C/HDL-C and LDL-C/ox-LDL →.	Farzin, 2020 [69]
Nonobese women randomized into placebo (*n* = 14) and RSV (*n* = 15) groups	75 mg/day, 12 weeks	intra-abdominal fat volume, intrahepatic triglyceride content, plasma lipids, and insulin sensitivity in the liver, skeletal muscle and adipose tissue →.	Yoshino, 2012 [70]
Healthy participants randomized into RSV (*n* = 24) and caloric restriction (*n* = 24) groups	500 mg/day, 30 days	plasma TG, HDL-C, LDL-C, and apolipoprotein A1 →, plasma TC and non-HDL cholesterol ↑.	Mansur, 2017 [71]
Patients with carotid stenosis >70% and in a surgical intervention request randomized to Cardioaspirin^®^ and Aterofisiol^®^ (*n* = 107) and Cardioaspirin^®^ and placebo (*n* = 107) groups	containing 20 mg RSV/day, 25 days	dry weight of lipid and cholesterol in removed plaques ↓ (0.232 ± 0.018 vs. 0.356 ± 0.022; 0.036 ± 0.006 vs. 0.053 ± 0.007 mg/mg dry weight, respectively).	Amato, 2015 [72]
Patients with type 2 diabetes mellitus and coronary heart disease randomized into placebo (*n* = 28) and RSV (*n* = 28) groups	500 mg/day, 4 weeks	plasma HDL-C ↑; plasma TC/HDL-C ↓; plasma TG, TC and LDL-C →.	Hoseini, 2019 [73]
Patients with stable coronary artery disease randomized into placebo (*n* = 20) and RSV (*n* = 20) groups	10 mg/day, 3 months	plasma LDL-C ↓; FMD ↑; plasma TG, TC and LDL-C →.	Magyar, 2012 [74]
Patients with stable coronary artery disease randomized into placebo (*n* = 25), grape extract (*n* = 25) and RSV-containing grape extract (*n* = 25) groups	containing ~8 mg RSV/day for 6 months and ~16 mg RSV/day for following 6 months	plasma TC and non-HDL-C ↓; inflammation ↓.	To-mé-Carneiro, 2013 [75]
Patients with stable coronary artery disease (*n* = 10) treated with placebo and RSV	330 mg/day, 3 days	FMD in patients who had undergone coronary artery bypass grafting ↑; FMD in those who had undergone percutaneous coronary intervention →.	Diaz, 2020 [76]

TG: triglyceride; TC: total cholesterol; HDL-C: high-density lipoprotein cholesterol; ox-LDL: oxidized low-density lipoprotein; LDL-C: low-density lipoprotein cholesterol; FMD: flow-mediated dilatation. The down arrow represents decrease; the right arrow represents no changes; the up arrow represents increase.

**Table 2:** Summary of clinical trials involving the use of RSV in hypertension.

Cohort (No.)	Dose and Duration of RSV	Main Outcome after RSV Administration	First Author, Year, Reference
Overweight/obese individuals with untreated borderline hypertension (*n* = 19) treated with 30, 90, and 270 mg RSV doses and placebo	30, 90, and 270 mg, 1 h	FMD ↑ in a dose-dependent manner	Wong, 2011 [86]
Hypertensive patients (*n* = 24) treated with placebo and RSV	300 mg, acute supplementation	FMD in women and individuals with higher LDL-C ↑.	Marques, 2018 [87]
Obese but otherwise healthy adults (*n* = 28) treated with placebo and RSV	75 mg/day, 6 weeks	FMD ↑; blood pressure →.	Wong, 2013 [88]
Hypertensive individuals (*n* = 18) treated with placebo and isolated phytochemicals	containing ~60 mg RSV/day, 28 days	diastolic blood pressure ↓; diastolic blood pressure →.	Biesinger, 2016 [89]

FMD: flow-mediated dilatation. The up arrow represents increase; the right arrow represents no changes; the down arrow represents decrease.

**Table 3:** Summary of clinical trials involving the use of RSV in ischemia.

Cohort (No.)	Dose and Duration of RSV	Main Outcome after RSV Administration	First Author, Year, Reference
Ischemic stroke patients with a clearly defined time of onset randomized to r-tPA plus placebo (early onset-to-treatment time, *n* = 78; delayed onset-to-treatment time, *n* = 80) and r-tPA plus RSV (early onset-to-treatment time, *n* = 77; delayed onset-to-treatment time, *n* = 77) groups	2.5 mg RSV/kg of body weight (maximum 250 mg), simultaneously with r-tPA	treatment outcomes in patients receiving delayed r-tPA treatment ↑; plasma matrix metalloproteinase-2 and matrix metalloproteinase-9 in patients receiving early or delayed r-tPA treatment ↓.	Chen, 2016 [98]
Healthy individuals (*n* = 22) treated with placebo and 2 doses of RSV	250 and 500 mg/day, 45 min	cerebral blood flow during task performance ↑ in a dose-dependent manner	Kennedy, 2010 [99]
Patients with stable angina pectoris randomized to RSV (*n* = 29), calcium fructoborate (*n* = 29) and RSV plus calcium fructoborate (*n* = 29) groups	20 mg/day, 60 days	plasma high-sensitivity C reactive protein, *n*-terminal prohormone of brain natriuretic peptide, TC, TG and LDL-C ↓; plasma HDL-C ↑.	Militaru, 2013 [100]
Older people with peripheral artery disease randomized to placebo (*n* = 22), 125 mg of RSV (*n* = 21) and 500 mg of RSV (*n* = 23) groups	125 and 500 mg/day, 6 months	6-min walk distance →.	McDermott, 2017 [101]
Patients with peripheral artery disease randomized to plain old balloon angioplasty (*n* = 75) and RSV drug-coated balloon (*n* = 78) groups	containing 0.9 µg/mm^2^, 6 and 12 months	in-lesion late lumen loss at 6 months and target lesion revascularization at 12 months compared to plain old balloon angioplasty group ↓; censored walking distance ↑.	Tepe, 2017 [102]
Patients with peripheral artery disease randomized to plain old balloon angioplasty (*n* = 75) and RSV drug-coated balloon (*n* = 78) groups	containing 0.9 µg/mm^2^, 24 months	target lesion revascularization ↓ and walking distance ↑ compared to plain old balloon angioplasty group	Albrecht, 2018 [103]

r-tPA: recombinant tissue-type plasminogen activator; TC: total cholesterol; TG: triglyceride; LDL-C: low-density lipoprotein cholesterol; HDL-C: high-density lipoprotein cholesterol. The up arrow represents increase; the down arrow represents decrease; the right arrow represents no changes.

**Table 4:** Summary of clinical trials involving the use of RSV in vascular complications of metabolic disease.

Cohort (No.)	Dose and Duration of RSV	Main Outcome after RSV Administration	First Author, Year, Reference
Obese but otherwise healthy men (*n* = 10) treated with placebo and RSV	150 mg/day, 30 days	postprandial plasma glucagon responses ↓; fasting plasma glucagon, glucagon-like peptide-1 and glucose-dependent insulinotropic polypeptide levels →.	Knop, 2013 [108]
Patients with non-alcoholic fatty liver disease randomized into placebo (*n* = 30) and RSV (*n* = 30) groups	600 mg/day, 3 months	plasma TC, LDL-C, glucose, aspartate aminotransferase and alanine aminotransferase ↓; plasma insulin, TG and HDL-C →.	Chen, 2015 [109]
Patients with type 2 diabetes randomized into placebo (*n* = 38) and RSV (*n* = 38) groups	1000 mg/day, 8 weeks	plasma glucose ↓; plasma HDL-C ↑; TG, TC and LDL-C →.	Abdollahi, 2019 [110]
Individuals at high risk of cardiovascular disease randomized into placebo (*n* = 25), RSV-rich grape supplement (*n* = 25) and grape supplement lacking RSV (*n* = 25) groups	8 mg/day for 6 months; 16 mg/day for following 6 months	high-sensitivity C-reactive protein, tumor necrosis factor-α, plasminogen activator inhibitor type 1 and interleukin-6/interleukin-10 ratio ↓; anti-inflammatory interleukin-10 ↑.	To-mé-Carneiro, 2012 [111]
Obese individuals with nonalcoholic fatty liver disease randomized into placebo (*n* = 8) and RSV (*n* = 8) groups	1500 mg/day, 6 months	basal and insulin-mediated very low-density lipoprotein-TG secretion, oxidation and clearance rates →.	Poulsen, 2018 [112]
Patients with well-controlled type 2 diabetes (*n* = 17) treated with placebo and RSV	150 mg/day, 30 days	hepatic and peripheral insulin sensitivity and intrahepatic lipid content →.	Timmers, 2016 [113]
Patients with type 2 diabetes (*n* = 14) treated with placebo and RSV	1000 mg/day, 5 weeks	glucagon-like peptide 1 secretion and glycemic control →.	Thazhath, 2016 [114]
Older glucose-intolerant individuals (*n* = 30) treated with placebo and RSV	2–3 g/day, 6 weeks	reactive hyperemia index ↑, plasma lipid profiles and blood pressure →.	Pollack, 2017 [115]
Patients with fatty acid oxidation (*n* = 9) treated with placebo and RSV	1000 mg/day, 4 weeks	fatty acid oxidation and exercise capacity →.	Storgaard, 2022 [116]
Patients at risk of developing type 2 diabetes mellitus (*n* = 8) treated with placebo and RSV	150 mg/day, 34 days	arterial ^18^F-fluoroxyglucose uptake and arterial inflammation →.	Boswijk, 2022 [117]
Overweight and obese pediatric subjects randomized to placebo (*n* = 11) and antioxidant supplementation (*n* = 16) groups	containing 20 mg RSV/day, 6 months	post-occlusive release hyperemic delta flow at 6 months ↑.	Pecoraro, 2022 [118]
Older adults with abdominal obesity (*n* = 22) treated with placebo and RSV plus curcumin	containing 200 mg RSV, 30 min before consuming the high-fat meal	cumulative postprandial response of soluble vascular cell adhesion molecule-1 ↓; circulating inflammatory markers and adhesion molecules →.	Vors, 2018 [119]
Overweight and slightly obese individuals (*n* = 45) treated with placebo and RSV	150 mg/day, 4 weeks	plasma lipid profiles, glucose, insulin, and markers for inflammation and endothelial function →.	van der Made, 2015 [120]
Overweight and slightly obese individuals (*n* = 45) treated with placebo and RSV	150 mg/day, 4 weeks	plasma lipid profiles, glucose, insulin, and markers for inflammation and endothelial function in the fasting state or postprandial phase →.	van der Made, 2017 [121]
Overweight older individuals randomized into placebo (*n* = 10), 300 mg RSV (*n* = 10), and 1000 mg RSV (*n* = 9) groups	300 and 1000 mg/day, 90 days	soluble vascular cell adhesion molecule-1 and total plasminogen activator inhibitor ↑ in the 1000 mg RSV vs. 300 mg RSV and placebo groups	Mankowski, 2020 [122]

TC: total cholesterol; LDL-C: low-density lipoprotein cholesterol; TG: triglyceride; HDL-C: high-density lipoprotein cholesterol. The down arrow represents decrease; the right arrow represents no changes; the up arrow represents increase.

## Data Availability

The data presented in this study are available in supplementary material.

## Supplementary Materials

The following supporting information can be downloaded at: https://www.mdpi.com/article/10.3390/molecules27217524/s1, Table S1: The effects of RSV on plasma lipid profiles in preclinical studies [39,60,61,62,63,64].
