# Correction: Fan et al. Progress in the Preclinical and Clinical Study of Resveratrol for Vascular Metabolic Disease. *Molecules* 2022, *27*, 7524

**DOI:** 10.3390/molecules29204840

**Published:** 2024-10-12

**Authors:** Dongxiao Fan, Chenshu Liu, Zhongyu Zhang, Kan Huang, Tengyao Wang, Sifan Chen, Zilun Li

**Affiliations:** 1 Division of Vascular Surgery, The First Affiliated Hospital of Sun Yat-sen University, Guangzhou 510080, China; 2 National-Guangdong Joint Engineering Laboratory for Diagnosis and Treatment of Vascular Diseases, The First Affiliated Hospital of Sun Yat-sen University, Guangzhou 510080, China; 3 Guangdong Provincial Key Laboratory of Malignant Tumor Epigenetics and Gene Regulation, Guangdong-Hong Kong Joint Laboratory for RNA Medicine, Sun Yat-sen Memorial Hospital, Sun Yat-sen University, Guangzhou 510120, China; 4 Medical Research Center, Sun Yat-sen Memorial Hospital, Sun Yat-sen University, Guangzhou 510120, China

**Text Correction**

- Figure 1 of the original publication [1]: the figure legend “PPARα: peroxisome proliferator-activated receptor-gamma coactivator-1α” should be “PPARα: peroxisome proliferator-activated receptor α”.

**Original:** PPARα: peroxisome proliferator-activated receptor-gamma coactivator-1α.
**Correction:** PPARα: peroxisome proliferator-activated receptor α.

- The fourth paragraph of Section 2.1 in the original publication, “peroxisome proliferator-activated receptor-gamma coactivator-1α (PPARα)” should be “peroxisome proliferator-activated receptor-gamma coactivator-1α (PGC1α)”.

**Original:** peroxisome proliferator-activated receptor-gamma coactivator-1α (PPARα).
**Correction:** peroxisome proliferator-activated receptor-gamma coactivator-1α (PGC1α).

- In the final paragraph of Section 2.2, “SIRT1-mediated activation of PPARα and its coactivator peroxisome proliferator-activated receptor-gamma coactivator-1α (PGC1α)” should be “SIRT1-mediated activation of peroxisome proliferator-activated receptor α (PPARα) and its coactivator PGC1α”.

**Original:** SIRT1-mediated activation of PPARα and its coactivator peroxisome proliferator-activated receptor-gamma coactivator-1α (PGC1α).
**Correction:** SIRT1-mediated activation of peroxisome proliferator-activated receptor α (PPARα) and its coactivator PGC1α.

The authors state that the scientific conclusions are unaffected. This correction was approved by the Academic Editor. The original publication has also been updated.
