# Supplementary material for: Progress in the Preclinical and Clinical Study of Resveratrol for Vascular Metabolic Disease
Source: Molecules. 2022 Nov 3;27(21):7524. doi: 10.3390/molecules27217524 (PMC9658204; doi:10.3390/molecules27217524)
Supplement: Supplementary file 1 [file molecules-27-07524-s001.zip › molecules-1812946-supplementary.pdf]

Supplementary Materials

Table S1. The effects of RSV on plasma lipid profiles in preclinical studies.

animal model	dose and duration of RSV	main results	first author, year, reference
Wistar rats treated with normal or 5% cholesterol diet	20 mg/kg, 20 days	RSV supplement significantly lowered plasma TC, TG, LDL-C, and VLDL-C levels and increased HDL-C levels (104.99 ± 1.63 vs. 66.5 ± 0.97, 196.00 ± 7.37 vs. 101.35 ± 3.38, 48.09 ± 0.77 vs. 26.38 ± 0.32, 39.28 ± 1.44 vs. 20.27 ± 0.68, and 17.41 ± 0.39 vs. 19.85 ± 0.74 mg/dL in control vs. RSV groups respectively) in cholesterol diet rat.	Göçmen, 2011, [1]
streptozotocin induced gestational diabetes model rats	60, 120 or 240 mg/kg, 2 weeks	RSV supplement decreased plasma TC, TG, and LDL-C and increased HDL-C in a dose dependent manner.	Zhang, 2021, [2]
KKAy mouse model	2 or 4 g/kg, 12 weeks	High dose of RSV supplement significantly reduced plasma TG and free fatty acid, and increased HDL-C.	Zhu, 2014, [3]
apoE-deficient mice fed with atherogenic diet	0.02% RSV in the diet, 12 weeks	RSV supplement significantly reduced plasma TG, TC, LDL-C, and non-HDL-C and increased HDL-C (1.07 ± 0.11 vs. 0.82 ± 0.07, 38.85 ± 1.36 vs. 29.44 ± 0.75, 34.01 ± 1.02 vs. 28.99 ± 0.62, 37.85 ± 1.45 vs. 28.72 ± 0.84, and 0.15 ± 0.03 vs. 0.28 ± 0.03 mM in control vs. RSV groups respectively).	Jeon, 2014, [4]
high fat diet fed mice	15 mg/kg, 10 weeks	RSV supplement reduced TG significantly in high fat diet fed mice.	Gong, 2020, [5]
healthy crossbred pigs	600 mg/kg, 119 days	RSV supplement significantly reduced plasma TG, TC, LDL-C and VLDL (2.96 ± 0.28 vs. 2.16 ± 0.14, 3.70 ± 0.30 vs. 2.89 ± 0.21, 1.99 ± 0.21 vs. 1.33 ± 0.17, and 17.87 ± 1.73 vs. 13.39 ± 0.48 mmol/gprot in control vs. RSV groups respectively).	Zhang, 2019, [6]

RSV: resveratrol; TC: total cholesterol; TG: triglyceride; LDL-C: low-density lipoprotein cholesterol; VLDL-C: very-low-density lipoprotein cholesterol; HDL-C: high-density lipoprotein cholesterol.

1. Göçmen, A.Y.; Burgucu, D.; Gümüşlü, S. Effect of resveratrol on platelet activation in hypercholesterolemic rats: CD40-CD40L system as a potential target. *Applied physiology, nutrition, and metabolism = Physiologie appliquee, nutrition et metabolisme* **2011**, *36*, 323-330, doi:10.1139/h11-022.
2. Zhang, G.; Wang, X.; Ren, B.; Zhao, Q.; Zhang, F. The Effect of Resveratrol on Blood Glucose and Blood Lipids in Rats with Gestational Diabetes Mellitus. *Evidence-based complementary and alternative medicine : eCAM* **2021**, *2021*, 2956795, doi:10.1155/2021/2956795.
3. Zhu, W.; Chen, S.; Li, Z.; Zhao, X.; Li, W.; Sun, Y.; Zhang, Z.; Ling, W.; Feng, X. Effects and mechanisms of resveratrol on the amelioration of oxidative stress and hepatic steatosis in KKAy mice. *Nutrition & metabolism* **2014**, *11*, 35, doi:10.1186/1743-7075-11-35.
4. Jeon, S.M.; Lee, S.A.; Choi, M.S. Antiobesity and vasoprotective effects of resveratrol in apoE-deficient mice. *Journal of medicinal food* **2014**, *17*, 310-316, doi:10.1089/jmf.2013.2885.
5. Gong, L.; Guo, S.; Zou, Z. Resveratrol ameliorates metabolic disorders and insulin resistance in high-fat diet-fed mice. *Life sciences* **2020**, *242*, 117212, doi:10.1016/j.lfs.2019.117212.
6. Zhang, H.Z.; Chen, D.W.; He, J.; Zheng, P.; Yu, J.; Mao, X.B.; Huang, Z.Q.; Luo, Y.H.; Luo, J.Q.; Yu, B. Long-term dietary resveratrol supplementation decreased serum lipids levels, improved intramuscular fat content, and changed the expression of several lipid metabolism-related miRNAs and genes in growing-finishing pigs1. *Journal of animal science* **2019**, *97*, 1745-1756, doi:10.1093/jas/skz057.
